# Telenursing in the postoperative period: a scoping review

**DOI:** 10.1590/0034-7167-2024-0066

**Published:** 2024-07-29

**Authors:** Viviane Cristina de Albuquerque Gimenez, Graziela Maria Ferraz de Almeida, Claudia Maria Silva Cyrino, Cassiane de Santana Lemos, Carolina Favoretto, Marla Andreia Garcia de Avila

**Affiliations:** I Universidade Estadual Paulista “Júlio de Mesquita Filho”. Botucatu, São Paulo, Brazil

**Keywords:** Telenursing, Telemonitoring, Surgical Procedures, Perioperative Nursing, Surgery, Teleasistencia, Telemonitorización, Procedimientos Quirúrgicos, Enfermería Perioperatoria, Cirugía

## Abstract

**Objectives:**

to map available evidence on telenursing use in the postoperative period and its impact on patient outcomes.

**Methods:**

a scoping review, conducted according to the JBI model and the PRISMA-ScR checklist. The search was carried out in the CINAHL, Embase, LILACS, PubMed, Web of Science, SciELO, Scopus and Cochrane Library databases.

**Results:**

twelve studies were included, published between 2011 and 2023, 66.6% of which were in developed countries. Of the positive outcomes, we highlight improved levels of disability, autonomy and quality of life, lower rates of post-operative complications, pain and reduced costs. Telephone monitoring was the most widely used modality, but there were few studies in the pediatric context and in Brazil.

**Conclusions:**

of the studies, 11 (91.6%) identified at least one positive outcome in telenursing use and none showed negative aspects in the postoperative period. The role of nurses in digital health needs further study.

## RESUMEN

**Objetivos:**

mapear la evidencia disponible sobre el uso de la teleasistencia en el periodo postoperatorio y su impacto en los resultados de los pacientes.

**Métodos:**

revisión de alcance, realizada según el modelo del JBI y la lista de verificación PRISMA-ScR. La búsqueda se realizó en las bases de datos CINAHL, Embase, LILACS, PubMed, Web of Science, SciELO, Scopus y Cochrane Library.

**Resultados:**

se incluyeron 12 estudios, publicados entre 2011 y 2023, de los cuales el 66,6% se realizaron en países desarrollados. Los resultados positivos incluyen mejora de los niveles de discapacidad, autonomía y calidad de vida, menores tasas de complicaciones postoperatorias, dolor y reducción de costes. La monitorización telefónica fue la modalidad más utilizada, pero hubo pocos estudios en el contexto pediátrico y en Brasil.

**Conclusiones:**

de los estudios, 11 (91,6%) identificaron al menos un resultado positivo en el uso de la teleenfermería y ninguno mostró aspectos negativos en el postoperatorio. El papel de las enfermeras en la salud digital requiere más estudios.

## INTRODUCTION

Remote care for users of healthcare services has been applied for various purposes, from screening to rehabilitation, reducing waiting times for appointments and travel costs. In this regard, it is possible to develop educational activities to prevent, monitor and control the symptoms of patients treated through this modality^(1)^.

Telemedicine, a term created in the 1970s, denotes “remote healing” and deviates from the traditional visit between a doctor and a patient^(2)^. According to Darkins and Cary, telemedicine is defined as the use of advanced telecommunications technologies with the aim of exchanging information and providing healthcare services in areas with geographic, temporal, social and cultural limitations^(3)^. Telehealth can be defined as any intervention in which clinical information is transferred remotely between patients and healthcare professionals^(4)^.

In the context of nursing, in mid-2004, several telenursing services in Canada, England and Wales already had the method implemented^(5)^ which, like telemedicine, is one of the methods of monitoring healthcare users with specific role of nurses.

Telehealth care, led by nurses, was expanded with the advent of the coronavirus disease (COVID-19) pandemic, which had as its strengths care provided without the risk of transmission of SARS-CoV-2, greater access to healthcare, continuous and patient-centered care and increased satisfaction among patients and nurses^(6)^.

In Brazil, during the COVID-19 pandemic, the Federal Nursing Council (COFEN - *Conselho Federal de Enfermagem*), through Resolution 634 of March 26, 2020, authorized and standardized nursing teleconsultation^(7)^ and, in 2022, the COFEN Resolution 696, amended by COFEN Resolutions 707/2022 and 717/2023, standardized the role of nursing in digital health within the scope of the Brazilian Health System as well as in supplementary health^(8-10)^.

The R esolution brings autonomy to nurses’ work in digital health and regulates telenursing practice encompasses nursing consultation, interconsultation, consultancy, monitoring, health education and acceptance of spontaneous demand as specific interventions mediated by the use of information and communication technologies (ICTs) and in accordance with the General Data Protection Law (GDPL)^(8-10)^.

Research conducted in Canada^(11)^, the United States^(12)^ and the Netherlands^(13)^ indicates the use of different strategies, such as telephone calls, text messages and video calls. Such strategies have been implemented by nurses to improve patient outcomes in the postoperative period, such as increased user satisfaction, reduced visits to hospital services due to lack of assistance, quality of care and reduced costs associated with the long distances traveled by patients to access in-person care^(11-13)^.

The relevance of the topic in the entire context of perioperative nursing stands out. However, some factors still need better understanding in their implementation stages, highlighting the different strategies used and their beneficial aspects for patients in the postoperative period.

## OBJECTIVES

To map the available evidence on the use of telenursing in the postoperative period and its impact on patient outcomes.

## METHODS

### Ethical aspects

The scoping review does not require a request for an opinion from a Research Ethics Committee, as it is a secondary study and does not directly involve human beings. Therefore, in this research, no ethical assessment was carried out, in accordance with Resolution 466/2012 of the Brazilian National Health Council.

### Study design, period and place

This is a scoping review, conducted in accordance with the JBI^(14)^ methodology for scoping reviews, whose purpose is to provide a map of evidence on a given topic, identify knowledge gaps, support the development of new studies as well as identify and clarify concepts/definitions used in the literature about a given object. For its execution, the following procedures were adopted: research question elaboration; inclusion and exclusion criteria definition; literature search and article selection; data analysis, synthesis and presentation^(14)^.

It is noteworthy that we included evidence and methodological quality assessment, although it is not recommended in scoping reviews.

The process of identifying articles until the final sample was synthesized and presented following the Preferred Reporting Items for Systematic reviews and Meta-Analyses extension for Scoping Reviews (PRISMA-ScR) recommendations^(15)^.

This is the first stage of study aimed at obtaining scientific foundations to develop and implement a telenursing protocol aimed at patients in the postoperative period of cranial surgery.

### Research question elaboration

The PCC^(14)^ strategy was used to develop the guiding question: Population (P): surgical patients; Concept (C): telenursing; Context (C): outcomes in the postoperative period.

Thus, the following research question was created: what evidence is available on the use of telenursing in the postoperative period and its impact on patient outcomes?

### Inclusion and exclusion criteria

We included (I) original articles related to the guiding question of the study, (II) covering adult and pediatric audiences, (III) in different surgical contexts.

We excluded (I) review studies, (II) qualitative studies, (III) cross-sectional studies, (IV) simple and expanded summaries, (V) posters, (VI) editorials, (VII) duplicate studies, (VIII) studies that started monitoring 30 days after surgery and (IX) studies not carried out by a nurse.

### Data search, selection, extraction and presentation of evidence

Study search was carried out in the Latin American and Caribbean Literature in Health Sciences (LILACS), National Center for Biotechonology Information (NCBI/PubMed), Web of Science (WoS), Cumulative Index to Nursing and Allied Health Literature (CINAHL), Scientific Electronic Library Online (SciELO), Scopus, Embase and Cochrane Library databases.

The strategies were constructed with the help of a librarian, adapted for each database, using descriptors and their synonyms: (Telenursing OR Telemonitoring OR “Remote Consultation” OR “Consultation, Remote” OR Teleconsultation OR Teleconsultations) AND (“Perioperative Nursing” OR “Surgical Nursing” OR “Nursing, Perioperative” OR “Perianesthesia Nursing” OR “Nursing, Perianesthesia” OR Nursing OR “Postanesthesia Nursing”) (Supplementary Material).

The studies were selected on a single day in August 2022. The reading took place from August to October 2022, and the search was reviewed in September 2023 in all databases, without fixing the year and language of publication, independently selected by two reviewers, using the Rayyan^®^ selection platform, developed by the Qatar Computing Research Institute (QCRI) as an auxiliary tool for archiving, organizing and selecting articles^(16)^.

In both moments of literature search, articles were selected after reading the titles and abstracts. Those that met the eligibility criteria and reached consensus between the two reviewers were read in full to define inclusion or exclusion from the review. Disagreements that occurred in the complete reading phase were discussed and resolved by a third reviewer.

### Research strategy, evidence selection and data extraction

To collect data from selected studies, the instruments proposed by the JBI model were used according to study design. A standardized form was adapted by the authors containing: author(s); year; country; language; study design; multicenter or single center; level of evidence; objective; age group; specialty; type of intervention; and outcomes. In light of the COFEN Resolution, studies were classified following the different telenursing modalities established in Brazil^(8-10)^.

### Analysis of evidence, presentation of results

To assess the classification of evidence from studies, the proposal by Melnyk and Fineout-Overholt was adopted^(17)^, which allows researchers to analyze different types of methods guided by the following criteria: I for systematic reviews and meta-analysis of randomized clinical trials; II for randomized clinical trials; III for non-randomized controlled trial; IV for case-control or cohort studies; V for systematic reviews of qualitative and/or descriptive studies; VI for qualitative or descriptive studies; VII for opinion from authorities and/or reports from expert committees. This hierarchy classifies levels I and II as strong, III to V as moderate, and VI to VII as weak.

### Methodological quality assessment

Two reviewers independently assessed the quality of the studies for risk of bias, and disagreements were resolved through consultation with a third reviewer. For cohort studies, we assessed study quality using the Newcastle-Ottawa Scale (NOS)^(18)^.

The NOS for cohort studies is composed of eight items. Each item can receive one point (one star), except the “comparability” item, whose score varies from zero to two stars. Low risk of study bias can receive a maximum score of nine stars for cohort studies. Cohort studies with six to eight stars were rated as moderate, and those with five stars or less were rated as low quality.

For clinical trials assessed, the risk of victory was assessed using Cochrane Collaboration^(19)^, according to the following criteria: generation of desired sequence (selection bias); allocation concealment (selection bias); blinding (performance bias and detection bias), considering blinding of participants and personnel and blinding of outcome assessment; incomplete outcome data (attrition bias); selective reporting (reporting bias); and other biases.

## RESULTS

**Figure 1:**
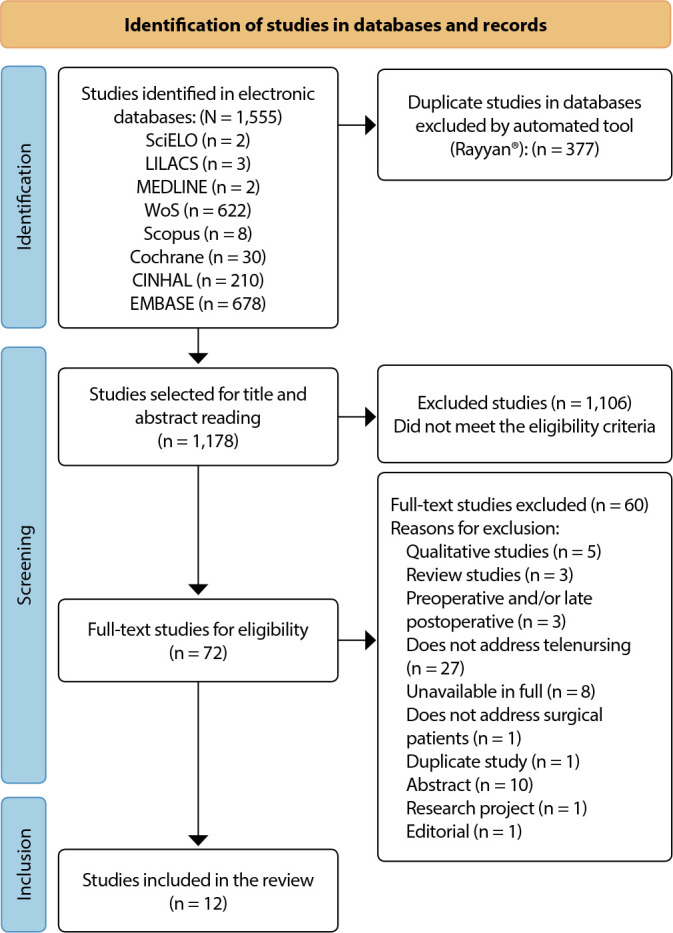
Flowchart of the selection and inclusion process of PRISMA-ScR studies - 2023

Through electronic database research, two references were identified in NCBI/PubMed, 210 in CINAHL Complete (via EBSCO), 622 in Web of Science, two in SciELO, three in LILACS, 678 in EMBASE, eight in Scopus and 30 in Cochrane, making a total of 1,555 studies. Of these, 377 were excluded due to duplication, 1,106 due to non-adherence to the inclusion criteria, and 72 were selected for full reading. Subsequently, 60 were excluded for not meeting the defined inclusion criteria. Finally, a total of 12 studies were included in this review, shown in Figure 1, based on the PRISMA-ScR flowchart^(15)^.

The studies were published between 2011 and 2023, of which nine (75%) were published in the last five years. The studies included were carried out in different countries, such as China (n=4; 33.3%)^(20-23)^, Turkey (n=2; 16.7%)^(24-25)^, United States of America (n=1; 8.3%)^(26)^, Canada (n=1; 8.3%)^(27)^, Brazil (n=1; 8.3%)^(1)^, Iran (n=1; 8.3%)^(28)^, Italy (n=1; 8.3%)^(29)^ and Australia (n=1; 8.3%)^(30)^, mostly available in English (n=11; 91.6%).

Regarding study design, 10 (83.3%) were characterized as randomized clinical trials, classified as level of evidence II, and cohort studies (n=2; 16.6%), classified as level of evidence IV. In relation to study execution, nine (75%) were carried out in single centers, and three (25%) in multicenter centers. The total population of all included studies was 2,098.

Of the surgical specialties, six (50%)^(1,20,22-23,28,30)^ studies performed telenursing in patients undergoing gastrosurgery; three (24.9%)^(25,27,29)^, in patients undergoing heart surgery; one (8.3%)^(26)^, in patients undergoing gynecological surgery; one (8.3%)^(21)^, in patients undergoing head and neck surgery; and one (8.3%)^(24)^, in patients undergoing neurosurgery. Only one (8.3%)^(20)^ study was developed with pediatric patients.

In light of the COFEN Resolution^(8-10)^, monitoring was the most frequently used type of telenursing (n=11; 91.6%), and one study carried out teleconsultation (8.3%). As interventions, telephone calls were predominant (n=9, 75%) among the studies; two (14.6%) used text messaging; and one (8.3%) associated telephone calls with video calls.

As evidenced outcomes, 11 (91.6%) studies reported better results in the telenursing intervention group, with emphasis on: improvement in disability levels, autonomy and activities of daily living, self-care skills, quality of life and improvement of knowledge in health (n=3)^(21-22,24-26)^; user satisfaction (n=3)^(1,23,26)^; stoma management (n=3)^(20,23,28)^; less need for acute care, lower rate of adverse events and postoperative complications (n=2)^(22,27)^; reduction in pain levels (n=2)^(24,27)^; improvement in anxiety and depression assessment scores, mental health levels (n=2)^(20,24)^; and cost reduction (n=1)^(22)^.

A (8.3%) study, which aimed to assess the impact of telenursing on symptoms and quality of life in patients undergoing esophageal and stomach resection surgery, correlated the comparison between intervention and control groups regarding symptoms, quality, distress, pain, admissions to emergency services and patient satisfaction. In this study, it was verified that, of the 81 patients assessed, there was no statistically significant difference between the control and intervention groups in relation to symptoms, quality of life, pain and distress during the proposed preand postoperative monitoring. Regarding search rates for emergency services and readmission, it was evident that there was a significant reduction in demand for the intervention group, although there was no statistical significance^(30)^ (Chart 1).

**Chart 1:** Identification, characterization, postoperative patient outcomes and type of telenursing, Botucatu, São Paulo, Brazil, 2024

Author/year/country/language	Study design/multicenter or single center/level of evidence	Objective	^*^IG versus †CG/age group/specialty/type of intervention		Outcomes						
Wu *et al*.^(20)^ 2023 China English	Retrospective cohort Single center IV	Compare the effects of continuous nursing using the WeChat^®^ platform with traditional nursing for post-enterostomy infants.	^*^IG: n=165 4.9±7.3 days	†CG: n=143 4.8±7.5 days	Peristomal skin DET scale ^*^IG: 2.3±1.4 †CG: 6.8±4.5 p=0.003	Replacing ostomy bags per week ^*^IG: 7.2±1.8 †CG: 18.5±3.5 p=0.002		Self-Rating Anxiety Scale (SAS) Three months after discharge ^*^IG: 48.1±11.3 †CG: 65.8±14.7 p=0.017			Self-Rating Depression Scale (SDS) ^*^IG: 40.1±9.6 †CG: 56.5±14.6 p=0.012
			Gastrosurgery WeChat^®^ platform (text messages) Monitoring								
Ding Y *et al*.^(21)^ 2022 China English	Randomized clinical trial Single center II	Compare telephone follow-up with traditional nurse-led follow-up structured according to the revised NOC.	^*^IG: n=51 <50 - > 70 years	†CG: n=49 <50 - > 70 years	Improvements in psychosocial health ^*^IG: 9.34±2.78 †CG: 4.80±3.09 p<0.001	Health knowledge/behavior ^*^IG: 12.52±9.27 †CG: 5.94±8.12 p<0.001		Changes in social conditions ^*^IG: 1.70±1.16 †CG: 0.62±0.83 p<0.001			Emotional health ^*^IG: 4.02±1.81 †CG: 1.22 ± 1.30 p<0.001
			Head and neck surgery (telephone call) Monitoring		Functional status ^*^IG: 2.12±1.56 †CG: 0.42±0.73 p<0.001	Additional attention ^*^IG: 2.06±1.57 †CG: 0.82±2.13 p=0.001		General self-care skills ^*^IG: 4.32±5.87 †CG: 2.40±0.62 p=0.016			Developed self-care skills ^*^IG: 3.70±2.41 †CG: 2.47±0.71 p<0.001
Lee DD *et al*.^(26)^ 2021 United States of America English	Randomized clinical trial Multicenter I	Determine patient satisfaction in virtual and traditional meetings in postoperative follow-up for pelvic organ prolapse.	^*^IG: n=26 59.9±10.9	†CG: n=26 58.0±11.3	^*^IG: Patient Satisfaction Questionnaire-18 (PSQ-18): 80.7 ± 2.6 Postoperative complication rates: 31%			†CG: Patient Satisfaction Questionnaire-18 (PSQ-18): 81.2 ± 2.8 Postoperative complication rates: 46%			
			Gynecological surgery (telephone call) Monitoring								
Chen K *et al*.^(22)^ 2021 China English	Cohort study Single center IV	Investigate the effectiveness of teleconsulting rehabilitation.	^*^IG: n=40 59.6±6.5	†CG: n=40 59.8±7.0	European Organization for Research and Treatment of Cancer Quality of Life (QoL) Questionnaire Core 30 (EORTC QLQ-C30) - Three months:			Esophageal Cancer Supplementary Scale (EORTC QLQ-OES18) Three months:			
			Gastrosurgery (telephone call) Teleconsulting		Sleep disorders ^*^IG: 47.50±24.91 †CG: 61.67±27.79 p=0.019			Difficulty swallowing saliva ^*^IG: 34.17±27.72 †CG: 51.67±31.08 p=0.010			
					Loss of appetite ^*^IG: 60.00±29.43 †CG: 76.67±21.62 p=0.005			Choking ^*^IG: 31.67±27.92 †CG: 45.83±27.93 p=0.024			
					Cost reduction ^*^IG: 56.67±20.26 †CG: 65.84±19.23 p=0.041			Coughing ^*^IG: 31.67±22.58 †CG: 43.33±27.43 p=0.041			
Aldemir and Gürkan^(24)^ 2021 Turkey English	Randomized clinical trial Single center II	Determine the effect of monitoring after herniated disc surgery on disability levels and quality of life.	^*^IG: n=33 29-65 years	†CG: n=34 31-64 years	36-Item Short Form Survey Social functionality ^*^IG: 84.47 ± 14.33 †CG: 66.18 ± 21.87 p=0.001		36-Item Short Form Survey Pain levels ^*^IG: 83.86 ± 19.33 †CG: 65.38 ± 28.80 p=0.003			36-Item Short Form Survey Mental health ^*^IG: 56.03 ± 6.98 †CG: 49.76 ± 11.32 p=0.008	
			Neurosurgery (telephone call) Monitoring		The Modified Oswestry Disability Index Level of disability in the third month ^*^IG: 3.45 (5.04) †CG: 6.65 (6.26) p=0.025		The pedometer and the Walking Program Application Chart Physical activity ^*^IG: 50.00 ± 34.79 †CG: 24.26 ± 26.46 p=0.001			The pedometer and the Walking Program Application Chart Energy and vitality ^*^IG: 58.33 ± 9.97 †CG: 46.76 ± 11.92 p=0.001	
Dığın F *et al*.^(25)^ 2021 Turkey English	Randomized clinical trial Single center II	Determine the effectiveness of telephone nursing consultations on the autonomy levels of elderly patients after myocardial revascularization.	^*^IG: n=32 ‡Me 69.96±4.94	†CG: n=32 ‡Me 69.96±4.94	Functional Autonomy Measurement System (SMAF) score Autonomy/resumption of activities of daily living ^*^IG: -2.20±1.71 †CG: -8.60±4.40 p=0.000						
			Cardiac surgery (telephone call) Monitoring								
McGillion MH *et al*.^(27)^ 2021 Canada English	Randomized clinical trial Multicenter II	Determine whether monitoring increases the length of stay of patients at home after non-elective surgeries when compared with standard care.	^*^IG: n=451 ‡Me 63.1 years	†CG: n=454 ‡Me 63.1 years	Medication error detection ^*^IG: 134 (29.7) †CG: 25 (5.5) p<0.001	Medication error correction ^*^IG: 128 (28.4) †CG: 18 (4.0) p<0.001		Pain on the 7^th^ day ^*^IG: 227/386 (58.8) †CG: 309/425 (72.7) p<0.001			Pain on the 15^th^ day ^*^IG: 193/402 (48.0) †CG: 248/414 (59.9) p<0.001
			Cardiac surgery (telephone call) Monitoring								
Oliveira DSS *et al*.^(1)^ 2021 Brazil Portuguese	Randomized clinical trial Single center II	Assess the impact of active nursing monitoring via telephone on the symptoms and quality of life of patients undergoing gastric surgery in outpatient follow-up.	^*^IG: n=41 Age not reported	†CG: n=40 Age not reported	EORTC QLQ-C30 Satisfaction						
			Gastrosurgery (telephone call) Monitoring		Good ^*^IG: 4 (12.5%) †CG: 2 (13.3%) p=0.002		Excellent ^*^IG: 28 (87.5%) †CG: 8 (53.3%) p=0.002			Never used ^*^IG: 0 (0.0%) †CG: 5 (33.3%) p=0.002	
Hamidi Y *et al*.^(28)^ 2018 Iran English	Randomized clinical trial Single center II	Assess the impact of an interactive follow-up program on the adaptation of ostomized patients after discharge.	^*^IG: n=32 18 to 80 years	†CG: n=32 18 to 80 years	Stoma adjustment before intervention ^*^IG: (5.83) 102.91 †CG: (5.78) 102.25 p< 0.001		Stoma adjustment immediately after the intervention ^*^IG: (7.68) 160.16 †CG: (6.96) 126.00 p< 0.001			Stoma adjustment one month after the intervention ^*^IG: (8.30) 161.41 †CG: (7.27) 152.56 p< 0.001	
			Gastrosurgery (telephone call) Monitoring								
Zhang J *et al*.^(23)^ 2013 China English	Randomized clinical trial Multicenter II	Analyze the effect of telephone monitoring by an ostomy specialist nurse on the adjustment levels of colostomized patients after hospital discharge.	^*^IG: n=52 ‡Me 52.9 years	†CG: n=51 ‡Me 55.3 years	Ostomy Adjustment Scale (OAS) Three months ^*^IG: 136.11 †CG: 124.32 p=0.006	Stoma Self-Efficacy Scale (SSES) Three months ^*^IG: 77.52 †CG: 70.02 p=0.014		Satisfaction with care Three months ^*^IG: 1.45 †CG: 2.04 p=0.000			Stoma complicationsThree months ^*^IG: 78.8% †CG: 56.9% p=0.044
			Oncology/gastrosurgery (telephone call) Monitoring								
Scalvini S *et al*.^(29)^ 2013 Italy English	Controlled clinical trial Single center II	Compare the ability to exercise in patients in the postoperative period of cardiac surgery with low to medium risk of mortality.	^*^IG: n=100 ‡Me 63	†CG: n=100 ‡Me 63	Hemoglobin (mg/dL) ^*^IG: 12.4 (1.2) †CG: 11.4 (1.2) p=0.001		Echocardiograms/ patient ^*^IG: 3.2 (3.0-3.4) †CG: 1.6 (1.4-1.8) p=0.001			Blood withdrawals/patient^*^IG: 5.6 (5.2-6.1) †CG: 7.1 (6.6-7.7) p=0.001	
			Cardiac surgery (Video call) (telephone call) Monitoring								
Harrison JD *et al*.^(30)^ 2011 Australia English	Randomized clinical trial Single center II	Determine the effectiveness of a nurse-delivered telephone support intervention.	^*^IG: n=38 ‡Me 67.2 years	†CG: n=36 ‡Me 61.8 years	Visits to emergency services ^*^IG: 21% †CG: 33% p=0.23				Hospital readmission ^*^IG:37% †CG: 47% p=0.37		
			Oncology/gastrosurgery (telephone call) Monitoring								

* *IG - intervention group; †CG - control group; ‡Me - mean.*

Charts 2 and 3 present the risk of bias assessment according to the specific scales Cochrane Collaboration^(19)^ for randomized clinical trial studies and modified NOS^(18)^ for cohort studies.

**Chart 2:** Assessment of the risk of bias in randomized clinical trials based on the Cochrane Collaboration Scale, Botucatu, São Paulo, Brazil, 2024

Author	Was allocation generation performed?	Was allocation concealment performed?	Has incomplete data control been checked?	Free from selective reporting of outcomes?	Were relevant outcomes assessed?	Were outcomes assessed with the investigator “blind” to the allocation groups?
Aldemir and Gürkan^*^	Yes	Yes	Yes	Yes	Yes	No
Digin F *et al*.^**^	Yes	Yes	No	Yes	Yes	No
McGillion MH *et al*.^*^	Yes	Yes	Yes	Yes	Yes	Yes
Lee DD *et al*.^*^	Yes	Yes	Yes	Yes	Yes	No
Oliveira DSS *et al*.^**^	Yes	NI	Yes	No	Yes	NI
Hamidi Y *et al*.^***^	Yes	No	No	Yes	Yes	No
Zhang J *et al*.^*^	Yes	Yes	Yes	Yes	Yes	Yes
Harrison JD *et al*.^*^	Yes	Yes	Yes	Yes	Yes	No
Ding Y *et al*.^**^	Yes	Yes	NI	Yes	Yes	NI
Scalvini S *et al*.^**^	Yes	No	NI	Yes	Yes	NI

NI - not informed;

* - Low risk of bias;

** - Risk of uncertain bias;

*** - High risk of bias.

**Chart 3:** Assessment of the risk of bias of cohort studies based on the Modified Newcastle-Ottawa Scale, Botucatu, São Paulo, Brazil, 2024

Author	Selection				Comparability	Outcomes			Total
	Representation of the exposed cohort	Selection of the unexposed cohort	Determination of exposure	Demonstration that the outcome of interest was not present at the beginning of the study	Comparability of cohorts based on design or analysis	Result assessment	Monitoring was long enough for results to occur	Adequacy of cohort monitoring	
Chen K *et al*.	C	a^*^	a^*^	a^*^	a^*^	a^*^	a^*^	a^*^	7^*^
Wu *et al*.	a^*^	a^*^	c	a^*^	a^*^	b^*^	a^*^	a^*^	7^*^

* *estrelas.*

Of the ten randomized clinical trials, five (50%) were considered to have a low risk of bias, four (40%) to have an uncertain risk of bias, and one (10%) to have a high risk of bias. Cohort studies presented seven stars, considered of moderate quality.

## DISCUSSION

As one of the telenursing actions, monitoring is identified as an innovative tool in the healthcare segment, which favors users’ accessibility to healthcare services, and should be used as a complement to healthcare, with the aim of promoting better access conditions, not replacing actions already carried out in person in healthcare services^(31)^.

Telenursing, in the post-operative period, proves to be a fruitful, innovative and challenging activity, which requires nurses to have scientific knowledge, technical skill and creativity, and can be used in the nursing process, in addition to the opportunity to promote health literacy. In this context, this study showed the predominance of monitoring as a strategy for providing care to post-operative patients at home. Among the main interventions, telephone calls^(1,21-27,29-30)^ and text messages^(20,28)^ stand out. The outcomes identified in the studies are diverse and also differ between the groups of surgeries and patients assessed.

As outcomes related to the surgical experience, telenursing favored self-care in the dimensions of general self-care skills, self-care skills developed and self-care skills developed with poor health status and total scores on the Appraisal of Self-Care Agency Scale^(21)^. Regarding the resumption of activities of daily living, telephone interventions brought significant results in the functional autonomy of elderly patients, measured by the Functional Autonomy Measurement System (SMAF)^(25)^. A randomized clinical trial, carried out in China, including 100 patients in the postoperative period of head and neck surgery, compared telephone monitoring versus traditional monitoring. The results show that telenursing increased health knowledge and behaviors (12.52±9.27 vs. 5.94±8.12; P<0.001^(21)^. A study conducted in Brazil considers that the telephone approach is a strategy for promoting health^(1)^_._

Telenursing has been shown to be beneficial for different patient outcomes in the postoperative period of lumbar disc hernia surgery. A randomized clinical trial with 33 patients in the intervention group and 34 in the control group aimed to compare the effect of walking supported by a pedometer through monitoring with a telephone call three weeks and after the completion of the first, second and third months postoperatively. The outcomes assessed were levels of pain, disability and quality of life. The results show that walking after surgery reduced pain and disability levels and increased quality of life^(24)^. Considering the pain outcome, patients in the intervention group showed a reduction in levels of sensory-perceptual pain, momentary pain and verbal pain measured by The Short Form McGill Pain Questionnaire (SF-MPQ) in the second and third months after surgery^(24)^.

Another study that assessed quality of life in 86 patients undergoing minimally invasive esophagectomy showed that telerehabilitation (rehabilitation guided by a messaging application called WeChat^®^) contributes to improving participants’ overall quality of life according to The European Organization for Research and Treatment of Cancer Quality of Life (QoL) Questionnaire Core 30 (EORTC QLQ-C30)^(22)^.

A multicenter randomized clinical trial, conducted in eight Canadian hospitals, including 905 post-operative patients from different surgeries, compared virtual care (telephone call and automated remote monitoring) versus in-person care for 31 days. It was found that participants in the virtual care group had lower levels of moderate to severe pain at 7 (58.8%), 15 (48%) and 30 (35%) days postoperatively. However, there were no differences between the groups when analyzing the effect of interventions on patient complaints and hospital readmissions^(24)^. An Australian pilot study, with six months of follow-up of 75 patients in the postoperative period of colorectal surgery, also indicates that telephone calls are effective in improving quality of life^(30)^.

User satisfaction was the outcome assessed in three studies^(1,23,26)^. Research conducted in Brazil, including 81 patients undergoing gastrosurgery, showed that 87.5% of patients rated the service provided by telephone monitoring as excellent versus 53.3% of patients in the control group (in-person service), with p=0.002^(1)^.

A multicenter study, using the Patient Satisfaction Questionnaire - 18 (PSQ-18), aimed to determine whether patient satisfaction in virtual meetings is not inferior to satisfaction in traditional in-person meetings for patients undergoing reconstructive surgery for pelvic organ prolapse. Fifty-two participants were included in the study and randomly assigned to two groups. The mean patient satisfaction score was 80.7 ± 2.6 in the virtual group and 81.2 ± 2.8 in the office group, consistent with non-inferiority. Postoperative complication rates were 31% in the virtual group and 46% in the office group. There were no significant differences between office visits, emergency room visits, and hospital readmissions within 90 days of surgery^(26)^.

A randomized clinical trial, developed in China, with 103 participants undergoing colostomy, showed that there was no significant difference between the control and intervention groups in the level of initial satisfaction. However, one month and three months after discharge, the intervention group showed significantly greater satisfaction^(23)^.

Other studies^(23,26)^ that assessed patient satisfaction in different surgical contexts presented similar results in relation to in-person and remote care, however the time factor was better assessed by participants. The time spent by users to go to the office, the waiting time for the service to begin and the time taken to return home were the main dissatisfactions reported. As a result, remote care duration, excluding travel time, was almost a third shorter compared to the group with in-person care^(23,26)^. A study focusing on cancer patients showed that telephone monitoring provided greater patient satisfaction in the intervention group, demonstrating the real impact of this process on cancer patient care^(1)^.

Supporting the study, the literature points to other benefits of telenursing associated with satisfaction, such as reducing barriers to accessing services, timely counseling and nursing care provided by nurses remotely, providing users with training to exercise control over their recovery process^(22-23,25,32-33)^.

Telenursing’s contribution to adequate use of emergency units by patients in the postoperative period is considered a still controversial outcome that needs to be better studied. A Brazilian study, monitoring patients with esophageal and stomach cancer for nine months postoperatively, found that, among the 40 patients in the control group and 41 patients in the intervention group, there was no reduction in the number of admissions to the emergency department^(1)^. Differently, a study carried out in the postoperative period of prostatectomy showed that patients in the intervention group showed a reduction in the search for emergency services when compared to the control group^(34)^. An Australian pilot study, with six months of follow-up of 75 patients in the postoperative period of colorectal surgery, also indicates that telephone calls are effective in reducing hospital readmission rates and demand for emergency services^(30)^.

In the present study, the specialty of gastrosurgery stands out, which was the object of study in six studies focusing on telenursing^(1,20,22-23,28,30)^, of which three^(20,23,28)^ addressed the surgeries to perform colostomy/jejunostomy. As for ostomy care, a clinical trial carried out in Iran, with 64 patients, found that, after one month of the intervention, with telephone calls and SMS messages, there was a significant difference between the two groups when comparing stoma adjustment and its dimensions (p<0.001)^(28)^. In line with this result, a randomized clinical trial, with 103 participants, carried out in China, assessed patients in relation to the Ostomy Adjustment Scale (OAS) and Stoma Self-Efficacy Scale (SSES). Participants in the intervention group, in the third month, had significantly better ostomy adjustment and greater stoma self-efficacy compared to the control group (p=0.006)^(23)^.

Regarding aspects related to mental health, a study assessed depression and anxiety three months after discharge from post-enterostomy surgery using the WeChat^®^ strategy (text messaging). Using self-assessment instruments for anxiety (Self-Rating Anxiety Scale (SAS)) (p=0.017) and depression (Self-Rating Depression Scale (SDS)) (p=0.012), the results show that the intervention group presented a statistically significant difference in the SAS and SDS scores when compared to the control group^(20)^. Research that analyzed, for six months, the effects of monitoring on 119 patients undergoing permanent colostomy, found that both groups presented better SAS and SDS scores post-intervention when compared to pre-intervention (the control group presented pre-intervention SAS of 61.02±7.48 and post-intervention of 53.38±6.12 and pre-intervention SDS of 60.29±7.21 and post-intervention of 52.07±4.26; the observation group presented SAS pre-intervention of 61.82±6.21 and post-intervention of 49.83±5.44 and SDS pre-intervention of 59.87±6.44 and post-intervention of 47.96±4.79)^(35)^.

One gap identified is the carrying out of studies including the pediatric population. A single study carried out with infants, a retrospective cohort, with 308 patients, assessed discoloration, erosion, erosion and tissue overgrowth (DET), proving that the intervention group had a lower DET score (p=0.003) and required fewer colostomy bag replacements three months after hospital discharge (p=0.002) when compared to the control group^(20)^.

Although telenursing shows promise for monitoring surgical patients, there is a need for further investigation, considering different scenarios and methodological rigor in carrying out studies. This modality favors access to information and, consequently, provides better health results. Discussion between peers enables support between specialists, which promotes increasingly assertive action, reducing the risk of harm resulting from healthcare and favoring significant learning for the nurses involved^(36)^.

It is also worth highlighting the scarcity of studies conducted in Brazil, a gap that must be filled in light of COFEN Resolution 696/2022^(8-9)^.

Finally, it is observed that no study was aimed at patients in the postoperative period of cranial surgery, signaling the importance of the study being developed by researchers who are implementing a protocol for monitoring via video call aimed at adult and pediatric patients in the postoperative period of neurosurgery.

### Study limitations

As a limitation of this study, we highlighted the exclusion of review, qualitative, editorial, theses and dissertations studies. We also do not register with the Open Science Framework (OSF). Furthermore, the quality of the monitoring protocol used in the studies was not assessed, an extremely relevant aspect that could interfere with the outcomes.

### Contributions to nursing, health, or public policies

Telenursing is a health technology that can further strengthen the autonomy of the nursing professional, highlighting their competence and relevance in the multidisciplinary team for making assertive decisions and a significant contribution to health promotion, prevention, and control of diseases, whether in individual or collective settings.

## CONCLUSIONS

Of the studies identified in this review, none presented negative aspects related to the use of telenursing and 11 (91.6%) identified at least one outcome with statistical significance in the use of telenursing versus in-person care in healthcare services for patients in the postoperative period. The outcomes identified were improvement in disability levels, autonomy and activities of daily living, self-care skills, quality of life, improvement of health knowledge, user satisfaction, stoma management, reduced need for acute care, lower rate of adverse events and postoperative complications, reduced pain levels, improved anxiety and depression assessment scores, mental health levels and reduced costs.

Telenursing was most used in developed countries, and monitoring was the most used modality for assessing patients postoperatively.

In this way, a promising and relevant scenario for nurses’ work is observed, with the need to develop new studies that include the development of protocols that meet the needs of each service/specialty and the beneficial aspects in quality of care in the postoperative period.
