# Supplementary material for: Telenursing in the postoperative period: a scoping review
Source: Rev Bras Enferm. 2024 Jul 29;77(3):e20240066. doi: 10.1590/0034-7167-2024-0066 (PMC11290728; doi:10.1590/0034-7167-2024-0066)
Supplement: Translation

# Translation: Telenfermagem no pós-operatório: revisão de escopo

**DOI:** 10.1590/0034-7167-2024-0066pt

**Authors:** Viviane Cristina de Albuquerque Gimenez, Graziela Maria Ferraz de Almeida, Claudia Maria Silva Cyrino, Cassiane de Santana Lemos, Carolina Favoretto, Marla Andreia Garcia de Avila

**Affiliations:** I Universidade Estadual Paulista “Júlio de Mesquita Filho”. Botucatu, São Paulo, Brasil

**Keywords:** Telenfermagem, Telemonitoramento, Procedimentos Cirúrgicos Operatórios, Enfermagem Perioperatória, Cirurgia

## Abstract

**Objetivos:**

mapear as evidências disponíveis sobre a utilização da telenfermagem no pós-operatório e seu impacto nos desfechos de pacientes.

**Métodos:**

revisão de escopo, conduzida de acordo com o modelo JBI e o *checklist* PRISMA-ScR. A busca foi realizada nas bases de dados CINAHL, Embase, LILACS, PubMed, *Web of Science*, SciELO, Scopus e *Cochrane Library*.

**Resultados:**

foram incluídos 12 estudos, publicados no período de 2011 a 2023, dos quais 66,6% foram em países desenvolvidos. Dos desfechos positivos, destacam-se melhora nos níveis de incapacidade, autonomia e qualidade de vida, menor taxa de complicações pós-operatórias, dor e redução de custos. O monitoramento por telefone foi a modalidade mais utilizada com escassez de produções no contexto pediátrico e no Brasil.

**Conclusões:**

dos estudos, 11 (91,6%) identificaram pelo menos um desfecho positivo no uso da telenfermagem e nenhum apresentou aspectos negativos no pós-operatório. A atuação do enfermeiro na saúde digital necessita de novos estudos.

## INTRODUÇÃO

O atendimento remoto aos usuários dos serviços de saúde tem sido aplicado para diversas finalidades, da triagem à reabilitação, reduzindo o tempo de espera para consultas e custos com deslocamento. Nesse sentido, é possível o desenvolvimento de atividades educativas de prevenção, acompanhamento e controle dos sintomas dos pacientes atendidos por meio dessa modalidade^(1)^.

A telemedicina, termo concebido na década de 1970, denota “cura a distância” e se desvia da tradicional visita do médico ao paciente^(2)^. Segundo Darkins e Cary, a telemedicina é definida como o uso de tecnologias avançadas de telecomunicações, com o intuito de trocar informações e fornecer serviços de saúde em áreas com limitações geográficas, temporais, sociais e culturais^(3)^. Já a telessaúde pode ser definida como qualquer intervenção em que a informação clínica é transferida remotamente entre o paciente e o profissional de saúde^(4)^.

No contexto da enfermagem, em meados de 2004, diversos serviços de telenfermagem no Canadá, na Inglaterra e no País de Gales já possuíam o método implementado^(5)^ que, assim como a telemedicina, é um dos métodos de acompanhamento dos usuários de saúde com atuação específica do enfermeiro.

O atendimento de telessaúde, conduzido por enfermeiros, foi ampliado com o advento da pandemia do *coronavirus disease* (covid-19), que teve como pontos fortes cuidados prestados sem o risco de transmissão da SARS-CoV-2, maior acesso aos cuidados de saúde, cuidado contínuo e centrado no paciente e aumento da satisfação entre pacientes e enfermeiros^(6)^.

No Brasil, durante a pandemia de covid-19, o Conselho Federal de Enfermagem (COFEN), por meio da Resolução nº 634, de 26 de março de 2020, autorizou e normatizou a teleconsulta de enfermagem^(7)^ e, no ano de 2022, a Resolução COFEN nº 696, alterada pelas Resoluções COFEN nº 707/2022 e nº 717/2023, normatizou a atuação da enfermagem na saúde digital no âmbito do Sistema Único de Saúde, bem como na saúde suplementar^(8-10)^.

A Resolução traz autonomia para a atuação do enfermeiro em saúde digital e regulamenta que a prática da telenfermagem engloba a consulta de enfermagem, a interconsulta, a consultoria, o monitoramento, a educação em saúde e o acolhimento da demanda espontânea como intervenções específicas, mediadas pelo uso de tecnologias de informação, comunicação (TICs) e consoante com a Lei Geral de Proteção de Dados (LGPD)^(8-10)^.

Pesquisas conduzidas no Canadá^(11)^, Estados Unidos^(12)^ e Holanda^(13)^ sinalizam a utilização de diferentes estratégias, como ligação telefônica, mensagens de texto e chamadas de vídeo. Tais estratégias têm sido implementadas por enfermeiros no sentido de melhorar os desfechos dos pacientes no período pós-operatório, como aumento da satisfação dos usuários, redução das idas aos serviços hospitalares em decorrência da falta de assistência, qualidade de cuidado e redução de custos associados às grandes distâncias percorridas pelos pacientes para ter acesso ao atendimento presencial^(11-13)^.

Destaca-se a relevância da temática em todo o contexto da enfermagem perioperatória. No entanto, alguns fatores ainda precisam de melhor compreensão nas suas etapas de implementação, destacando-se as diferentes estratégias utilizadas e seus aspectos benéficos para os pacientes no pós-operatório.

## OBJETIVOS

Mapear as evidências disponíveis sobre a utilização da telenfermagem no pós-operatório e seu impacto nos desfechos de pacientes.

## MÉTODOS

### Aspectos éticos

A revisão de escopo dispensa pedido de parecer a um Comitê de Ética em Pesquisa, por se tratar de estudo secundário e não envolver seres humanos diretamente. Por isso, nesta pesquisa, não foi realizada apreciação ética, conforme a Resolução nº 466/2012 do Conselho Nacional de Saúde.

### Desenho, período e local do estudo

Trata-se de revisão de escopo, conduzida de acordo com a metodologia JBI^(14)^ para revisões de escopo, cuja finalidade é fornecer um mapa das evidências sobre determinado tema, identificar lacunas do conhecimento, subsidiar o desenvolvimento de novos estudos, bem como identificar e esclarecer conceitos/definições utilizados na literatura sobre determinado objeto. Para sua execução, os seguintes procedimentos foram adotados: elaboração da pergunta de pesquisa; definição dos critérios de inclusão e exclusão; busca na literatura e seleção dos artigos; análise, síntese e apresentação dos dados^(14)^.

Destaca-se que incluímos a avaliação da qualidade de evidências e metodológica, embora não seja recomendado nas revisões de escopo.

O processo de identificação dos artigos até a amostra final foi sintetizado e apresentado seguindo as recomendações da *Preferred Reporting Items for Systematic reviews and Meta-Analyses extension for Scoping Reviews* (PRISMA-ScR)^(15)^.

Trata-se de primeira etapa de estudo que visa à obtenção de fundamentação científica para desenvolver e implementar um protocolo de telenfermagem direcionado aos pacientes no pós-operatório de cirurgia craniana.

### Elaboração da pergunta de pesquisa

A estratégia PCC^(14)^ foi utilizada para elaborar a questão norteadora: População (P): pacientes cirúrgicos; Conceito (C): telenfermagem; Contexto (C): desfechos no período pós-operatório.

Nesse sentido, a seguinte pergunta de investigação foi elaborada: quais as evidências disponíveis sobre a utilização da telenfermagem no pós-operatório e seu impacto nos desfechos de pacientes?

### Critérios de inclusão e exclusão

Foram incluídos (I) artigos originais relacionados à questão norteadora do estudo, (II) com abrangência do público adulto e pediátrico, (III) nos diferentes contextos cirúrgicos.

Foram excluídos (I) estudos de revisão, (II) estudos qualitativos, (III) estudos transversais, (IV) resumos simples e expandidos, (V) pôsteres, (VI) editoriais, (VII) estudos duplicados, (VIII) estudos que iniciaram o monitoramento 30 dias após a cirurgia e (IX) estudos não realizados por enfermeiro.

### Busca de dados, seleção, extração e apresentação das evidências

A busca dos estudos foi realizada nas bases de dados Literatura Latino-Americana e do Caribe em Ciências da Saúde (LILACS), *National Center for Biotechonology Information* (NCBI/PubMed), *Web of Science* (WoS), *Cumulative Index to Nursing and Allied Health Literature* (CINAHL), *Scientific Electronic Library Online* (SciELO), Scopus, Embase e *Cochrane Library*.

As estratégias foram construídas com auxílio de bibliotecária, adaptada para cada base de dados, utilizando os descritores e seus sinônimos: (*Telenursing OR Telemonitoring OR* “*Remote Consultation*” *OR* “*Consultation, Remote*” *OR Teleconsultation OR Teleconsultations*) *AND* (“*Perioperative Nursing*” *OR* “*Surgical Nursing*” *OR* “*Nursing, Perioperative*” *OR* “*Perianesthesia Nursing*” *OR* “*Nursing, Perianesthesia*” *OR Nursing OR* “*Postanesthesia Nursing*”) (Material Suplementar).

Os estudos foram selecionados em um único dia do mês de agosto de 2022. A leitura se deu no período de agosto a outubro de 2022, e a busca foi revisada no mês de setembro de 2023 em todas as bases, sem fixação de ano e idioma da publicação, selecionados de maneira independente por dois revisores, por meio da plataforma de seleção Rayyan^®^, desenvolvida pelo *Qatar Computing Research Institute* (QCRI) como uma ferramenta auxiliar para arquivar, organizar e selecionar artigos^(16)^.

Nos dois momentos de busca na literatura, os artigos foram selecionados após a leitura dos títulos e dos resumos. Os que atenderam aos critérios de elegibilidade e tiveram consenso entre os dois revisores foram lidos na íntegra para definir inclusão ou exclusão da revisão. As discordâncias que ocorreram na fase de leitura completa foram discutidas e resolvidas por um terceiro revisor.

### Estratégia de pesquisa, seleção de evidências e extração dos dados

Para coletar os dados dos estudos selecionados, foram utilizados os instrumentos propostos pelo modelo da JBI de acordo com o tipo de estudo. Um formulário padronizado foi adaptado pelos autores contendo: autor(es); ano; país; idioma; desenho do estudo; multicêntrico ou centro único; nível de evidência; objetivo; faixa etária; especialidade; tipo de intervenção; e desfechos. À luz da Resolução COFEN, realizou-se a classificação dos estudos seguindo as diferentes modalidades de telenfermagem estabelecidas no Brasil^(8-10)^.

### Análise das evidências, apresentação dos resultados

Para avaliar a classificação de evidências dos estudos, foi adotada a proposta de Melnyk e Fineout-Overholt^(17)^, que possibilita ao pesquisador analisar diferentes tipos de métodos norteados pelos seguintes critérios: I para revisões sistemáticas e metanálise de ensaios clínicos randomizados; II para ensaios clínicos randomizados; III para ensaio controlado não randomizado; IV para estudos caso-controle ou coorte; V para revisões sistemáticas de estudos qualitativos e/ou descritivos; VI para estudos qualitativos ou descritivos; VII para parecer de autoridades e/ou relatórios de comitês de especialistas. Essa hierarquia classifica os níveis I e II como fortes, III a V, como moderados, e VI a VII, como fracos.

### Avaliação da qualidade metodológica

Dois revisores avaliaram independentemente a qualidade dos estudos quanto ao risco de viés, e as divergências foram resolvidas por meio de consulta a um terceiro revisor. Para estudos de coorte, avaliamos a qualidade dos estudos utilizando a Escala de Newcastle-Ottawa (NOS)^(18)^.

A NOS para estudos de coorte é composta de oito itens. Cada item pode receber um ponto (uma estrela), exceto o item “comparabilidade”, cuja pontuação varia de zero a duas estrelas. O baixo risco de viés de estudos pode receber uma pontuação máxima de nove estrelas para estudos de coorte. Estudos de coorte de seis a oito estrelas foram classificados como moderados, e os com cinco estrelas ou menos foram considerados como de baixa qualidade.

Para ensaios clínicos randomizados, o risco de viés foi avaliado por meio da ferramenta *Cochrane Collaboration* ^(19)^, de acordo com os seguintes critérios: geração de sequência aleatória (viés de seleção); ocultação de alocação (viés de seleção); cegamento (viés de desempenho e viés de detecção), considerando cegamento de participantes e pessoal e cegamento da avaliação de resultados; dados de resultados incompletos (viés de atrito); relatórios seletivos (viés de relato); e outros vieses.

## RESULTADOS

Foram identificadas, por meio da pesquisa eletrônica em bases de dados, duas referências na NCBI/PubMed, 210 na CINAHL *Complete* (via EBSCO), 622 na *Web of Science*, duas na SciELO, três na LILACS, 678 na EMBASE, oito na Scopus e 30 na Cochrane, perfazendo um total de 1.555 estudos. Desses, 377 foram excluídos por duplicidade, 1.106, por não adesão aos critérios de inclusão, e 72 foram selecionados para leitura na íntegra. Na sequência, 60 foram excluídos por não cumprirem os critérios de inclusão definidos. Por fim, foram incluídos nesta revisão um total de 12 estudos, demonstrados na Figura 1, a partir do fluxograma PRISMA-ScR^(15)^.

**Figura 1:** Fluxograma do processo de seleção e inclusão de estudos PRISMA-ScR - 2023

Os estudos foram publicados no período de 2011 a 2023, dos quais nove (75%) foram publicados nos últimos cinco anos. Os estudos incluídos foram realizados em diferentes países, como China (n=4; 33,3%)^(20-23)^, Turquia (n=2; 16,7%)^(24-25)^, Estados Unidos da América (n=1; 8,3%)^(26)^, Canadá (n=1; 8,3%)^(27)^, Brasil (n=1; 8,3%)^(1)^, Irã (n=1; 8,3%)^(28)^, Itália (n=1; 8,3%)^(29)^ e Austrália (n=1; 8,3%)^(30)^, disponíveis majoritariamente no idioma inglês (n=11; 91,6%).

Quanto ao tipo de estudo, 10 (83,3%) caracterizavam-se como ensaios clínicos randomizados, classificados com nível de evidência II, e estudos de coorte (n=2; 16,6%), classificados com nível de evidência IV. Quanto à execução do estudo, nove (75%) foram realizados em centros únicos, e três, em (25%) centros multicêntricos. A população total de todos os estudos incluídos foi de 2.098.

Das especialidades cirúrgicas, seis (50%)^(1,20,22-23,28,30)^ estudos realizaram a telenfermagem em pacientes submetidos à gastrocirurgia; três (24,9%)^(25,27,29)^, em pacientes submetidos à cirurgia cardíaca; um (8,3%)^(26)^, em pacientes submetidos à cirurgia ginecológica; um (8,3%)^(21)^, em pacientes submetidos à cirurgia de cabeça e pescoço; e um (8,3%)^(24)^, em pacientes submetidos à neurocirurgia. Apenas um (8,3%)^(20)^ estudo foi desenvolvido com o paciente pediátrico.

À luz da Resolução COFEN^(8-10)^, o monitoramento foi a modalidade de telenfermagem mais realizada (n=11; 91,6%), e um estudo realizou teleconsultoria (8,3%). Como intervenções, a ligação telefônica foi predominante (n=9, 75%) entre os estudos; dois (14,6%) utilizaram a mensagem de texto; e um (8,3%) associou a ligação telefônica com chamadas de vídeo.

Como desfechos evidenciados, 11 (91,6%) estudos relataram melhores resultados no grupo de intervenção de telenfermagem, com destaque para: melhora dos níveis de incapacidade, autonomia e atividades da vida diária, habilidades do autocuidado, qualidade de vida e aprimoramento do conhecimento em saúde (n=3)^(21-22,24-26)^; satisfação do usuário (n=3)^(1,23,26)^; manejo de estomas (n=3)^(20,23,28)^; menor necessidade de cuidados agudos, menor taxa de eventos adversos e complicações pós-operatórias (n=2)^(26-27)^; redução dos níveis de dor (n=2)^(24,27)^; melhora nos escores de avaliação de ansiedade e depressão, níveis de saúde mental (n=2)^(20,24)^; e redução de custos (n=1)^(22)^.

Um (8,3%) estudo, que objetivou avaliar o impacto da telenfermagem nos sintomas e na qualidade de vida em pacientes submetidos à cirurgia de ressecção de esôfago e estômago, correlacionou a comparação entre grupo intervenção e controle quanto à sintomatologia, qualidade, sofrimento, dor, admissões nos serviços de emergência e satisfação dos pacientes. Foi verificado, neste estudo, que, dos 81 pacientes avaliados, não houve diferença estatisticamente significante entre os grupos controle e intervenção em relação à sintomatologia, qualidade de vida, dor e sofrimento durante o acompanhamento proposto no pré e pós-operatório. Quanto às taxas de busca por serviços de emergência e reinternação, evidenciou-se que houve redução importante da procura pelo grupo intervenção, apesar de não se verificar significância estatística^(30)^ (Quadro 1).

**Quadro 1:** Identificação, caracterização, desfechos dos pacientes no pós-operatório e tipo de telenfermagem, Botucatu, São Paulo, Brasil, 2024

Autor/ano/país/idioma	Desenho do estudo/multicêntrico ou centro único/nível de evidência	Objetivo	^*^GI *versus* †GC/ faixa etária/ especialidade/ tipo de intervenção		Desfechos					
Wu *et al*.^(20)^ 2023 China Inglês	Coorte retrospectivo Centro único IV	Comparar os efeitos da enfermagem contínua utilizando a plataforma *WeChat^®^* com a enfermagem tradicional para neonatos pós-enterostomia.	^*^GI: n=165 4,9±7,3 dias	†GC: n=143 4,8±7,5 dias	*Peristomal skin DET scale* ^*^GI: 2,3±1,4 †GC: 6,8±4,5 p=0,003	Substituição de bolsas de ostomia por semana ^*^GI: 7,2±1,8 †GC: 18,5±3,5 p=0,002		*Self-Rating Anxiety Scale* (SAS) Três meses após alta ^*^GI: 48,1±11,3 †GC: 65,8±14,7 p=0,017		*Self-Rating Depression Scale* (SDS) ^*^GI: 40,1±9,6 †GC: 56,5±14,6 p=0,012
			Gastrocirurgia Plataforma *WeChat^®^* (mensagens de texto) Monitoramento							
Ding Y *et al*.^(21)^ 2022 China Inglês	Ensaio clínico randomizado Centro único II	Comparar o acompanhamento telefônico com o acompanhamento tradicional liderado por enfermeiras, estruturado de acordo com a NOC revisada.	^*^GI n=51 <50 - > 70 anos	†GC n=49 <50 - > 70 anos	Melhorias na saúde psicossocial ^*^GI: 9,34±2,78 †GC: 4,80±3,09 p<0,001	Conhecimento/comportamento em saúde ^*^GI: 12,52±9,27 †GC: 5,94±8,12 p<0,001		Mudanças nas condições sociais ^*^GI: 1,70±1,16 †GC: 0,62±0,83 p<0,001		Saúde emocional ^*^GI: 4,02±1,81 †GC: 1,22 ± 1,30 p<0,001
			Cirurgia de cabeça e pescoço (ligação telefônica) Monitoramento		Estado funcional ^*^GI: 2,12±1,56 †GC: 0,42±0,73 p<0,001	Atenção adicional ^*^GI: 2,06±1,57 †GC: 0,82±2,13 p=0,001		Habilidades gerais de autocuidado ^*^GI: 4,32±5,87 †GC: 2,40±0,62 p=0,016		Habilidades de autocuidado desenvolvidas ^*^GI: 3,70±2,41 †GC: 2,47±0,71 p<0,001
Lee DD *et al*.^(26)^ 2021 Estados Unidos da América Inglês	Ensaio clínico randomizado Multicêntrico I	Determinar a satisfação do paciente em encontros virtuais e tradicionais em acompanhamento pós-operatório de prolapso de órgãos pélvicos.	^*^GI: n=26 59,9±10,9	†GC: n=26 58,0±11,3	^*^GI: *Patient Satisfaction Questionnaire-*18 (PSQ-18): 80,7 ± 2,6 Taxas de complicações pós-operatórias: 31%			†GC: *Patient Satisfaction Questionnaire*-18 (PSQ-18): 81,2 ± 2,8 Taxas de complicações pós-operatórias: 46%		
			Cirurgia ginecológica (ligação telefônica) Monitoramento							
Chen K *et al*.^(22)^ 2021 China Inglês	Estudo de coorte Centro único IV	Investigar a eficácia da reabilitação por teleconsultoria.	^*^GI: n=40 59,6±6,5	†GC: n=40 59,8±7,0	*European Organization for Research and Treatment of Cancer Quality of Life* (QoL) *Questionnaire Core* 30 (EORTC QLQ-C30) - três meses:			*Esophageal Cancer Supplementary Scale* (EORTC QLQ-OES18) três meses:		
			Gastrocirurgia (ligação telefônica) Teleconsultoria		Distúrbios do sono ^*^GI: 47,50±24,91 †GC: 61,67±27,79 p=0,019			Dificuldade na deglutição de saliva ^*^GI: 34,17±27,72 †GC: 51,67±31,08 p=0,010		
					Perda de apetite ^*^GI: 60,00±29,43 †GC: 76,67±21,62 p=0,005			Engasgo ^*^GI: 31,67±27,92 †GC: 45,83±27,93 p=0,024		
					Redução de custos ^*^GI: 56,67±20,26 †GC: 65,84±19,23 p=0,041			Tosse ^*^GI: 31,67±22,58 †GC: 43,33±27,43 p=0,041		
Aldemir e Gürkan^(24)^ 2021 Turquia Inglês	Ensaio clínico randomizado Centro único II	Determinar o efeito do monitoramento após cirurgia de hérnia de disco nos níveis de incapacidade e na qualidade de vida,	^*^GI: n=33 29-65 anos	†GC: n=34 31-64 anos	*36-Item Short Form Survey* Funcionalidade social ^*^GI: 84,47 ± 14,33 †GC: 66,18 ± 21,87 p=0,001		*36-Item Short Form Survey* Níveis de dor ^*^GI: 83,86 ± 19,33 †GC: 65,38 ± 28,80 p=0,003			*36-Item Short Form Survey* Saúde mental ^*^GI: 56,03 ± 6,98 †GC: 49,76 ± 11,32 p=0,008
			Neurocirurgia (ligação telefônica) Monitoramento		*The Modified Oswestry Disability Index* Nível de incapacidade no terceiro mês ^*^GI: 3,45 (5,04) †GC: 6,65 (6,26) p=0,025		*The pedometer and the Walking Program Application Chart* Atividade física ^*^GI: 50,00 ± 34,79 †GC: 24,26 ± 26,46 p=0,001			*The pedometer and the Walking Program Application Chart* Energia e vitalidade ^*^GI: 58,33 ± 9,97 †GC: 46,76 ± 11,92 p=0,001
Dığın F *et al*.^(25)^ 2021 Turquia Inglês	Ensaio Clínico Randomizado Centro Único II	Determinar a eficácia da consulta telefônica de enfermagem nos níveis de autonomia de pacientes idosos após revascularização do miocárdio.	^*^GI: n=32 ‡Me 69,96±4,94	†GC: n=32 ‡Me 69,96±4,94	*Functional Autonomy Measurement System* (SMAF) *score* Autonomia/retomada das atividades de vida diária ^*^GI: -2,20±1,71 †GC: -8,60±4,40 p=0,000					
			Cirurgia cardíaca (ligação telefônica) Monitoramento							
McGillion MH *et al*.^(27)^ 2021 Canadá Inglês	Ensaio clínico randomizado Multicêntrico II	Determinar se o monitoramento aumenta o tempo de permanência no domicílio de pacientes após cirurgias não eletivas, quando comparados com o atendimento padrão.	^*^GI: n=451 ‡Me 63,1 anos	†GC: n=454 ‡Me 63,1 anos	Detecção de erro de medicação ^*^GI: 134 (29,7) †GC: 25 (5,5) p<0,001	Correção de erro de medicação ^*^GI: 128 (28,4) †GC: 18 (4,0) p<0,001		Dor no 7º dia ^*^GI: 227/386 (58,8) †GC: 309/425 (72,7) p<0,001		Dor no 15º dia ^*^GI: 193/402 (48,0) †GC: 248/414 (59,9) p<0,001
			Cirurgia cardíaca (ligação telefônica) Monitoramento							
Oliveira DSS *et al*.^(1)^ 2021 Brasil Português	Ensaio clínico randomizado Centro único II	Avaliar o impacto do monitoramento ativo de enfermagem por telefone nos sintomas e na qualidade de vida de pacientes submetidos a cirurgias gástricas em seguimento ambulatorial.	^*^GI: n=41 Idade não informada	†GC: n=40 Idade não informada	EORTC QLQ-C30 Satisfação					
			Gastrocirurgia (ligação telefônica) Monitoramento		Bom ^*^GI: 4 (12,5%) †GC: 2 (13,3%) p=0,002		Excelente ^*^GI: 28 (87,5%) †GC: 8 (53,3%) p=0,002			Nunca utilizou ^*^GI: 0 (0,0%) †GC: 5 (33,3%) p=0,002
Hamidi Y *et al*.^(28)^ 2018 Irã Inglês	Ensaio clínico randomizado Centro único II	Avaliar o impacto de um programa de seguimento interativo na adaptação de pacientes ostomizados após a alta.	^*^GI: n=32 18 a 80 anos	†GC: n=32 18 a 80 anos	Ajuste do estoma antes da intervenção ^*^GI: (5,83) 102,91 †CG: (5,78) 102,25 p< 0,001		Ajuste do estoma imediatamente após a intervenção ^*^GI: (7,68) 160,16 †GC: (6,96) 126,00 p< 0,001			Ajuste do estoma um mês após a intervenção ^*^GI: (8,30) 161,41 †GC: (7,27) 152,56 p< 0,001
			Gastrocirurgia (mensagem de texto) Monitoramento							
Zhang J *et al*.^(23)^ 2013 China Inglês	Ensaio clínico randomizado Multicêntrico II	Analisar o efeito do acompanhamento telefônico da enfermeira especialista em ostomias nos níveis de ajuste de pacientes colostomizados após a alta hospitalar.	^*^GI: n=52 ‡Me 52,9 anos	†GC: n=51 ‡Me 55,3 anos	*Ostomy Adjustment Scale* (OAS) Três meses ^*^GI: 136,11 †GC: 124,32 p=0,006	*Stoma Self-Efficacy Scale* (SSES) Três meses ^*^GI: 77,52 †GC: 70,02 p=0,014		Satisfação com o cuidado Três meses ^*^GI: 1,45 †GC: 2,04 p=0,000		Complicações do estoma Três meses ^*^GI: 78,8% †GC: 56,9% p=0,044
			Oncologia/gastrocirurgia (ligação telefônica) Monitoramento							
Scalvini S *et al*.^(29)^ 2013 Itália Inglês	Ensaio clínico controlado Centro único II	Comparar a capacidade de realização de exercício em pacientes no pós-operatório de cirurgia cardíaca com baixo a médio risco de mortalidade.	^*^GI: n=100 ‡Me 63	†GC: n=100 ‡Me 63	Hemoglobina (mg/dL) ^*^GI: 12,4 (1,2) †GC: 11,4 (1,2) p=0,001		Ecocardiogramas/paciente ^*^GI: 3,2 (3,0-3,4) †GC: 1,6 (1,4-1,8) p=0,001			Retiradas de sangue/paciente ^*^GI: 5,6 (5,2-6,1) †GC: 7,1 (6,6-7,7) p=0,001
			Cirurgia cardíaca (chamada de vídeo) (ligação telefônica) Monitoramento							
Harrison JD *et al*.^(30)^ 2011 Austrália Inglês	Ensaio clínico randomizado Centro único II	Determinar a eficácia de uma intervenção de suporte telefônico fornecida por enfermeiro.	^*^GI: 38 ‡Me 67,2 anos	†GC: 36 ‡Me 61,8 anos	Idas aos serviços de urgência ^*^GI: 21% †GC: 33% p=0,23				Readmissão hospitalar ^*^GI:37% †GC: 47% p=0,37	
			Oncologia/gastrocirurgia (ligação telefônica) Monitoramento							

* *GI - grupo intervenção; †GC - grupo controle; ‡Me - média.*

Nos Quadros 2 e 3, é apresentada a avaliação do risco de viés, conforme as escalas específicas *Cochrane Collaboration* ^(19)^ para os estudos de ensaio clínico randomizado e NOS modificada^(18)^ para estudos de coorte.

**Quadro 2:** Avaliação do risco de viés de ensaios clínicos randomizados com base na Escala *Cochrane Collaboration*, Botucatu, São Paulo, Brasil, 2024

Autor	A geração de alocação foi realizada?	A ocultação da alocação foi realizada?	O controle de dados incompleto foi verificado?	Livre de relato seletivo de desfechos?	Os desfechos relevantes foram avaliados?	Os desfechos foram avaliados com o investigador “cego” para os grupos de alocação?
Aldemir and Gürkan^*^	Sim	Sim	Sim	Sim	Sim	Não
Digin F *et al*.^**^	Sim	Sim	Não	Sim	Sim	Não
McGillion MH *et al*.^*^	Sim	Sim	Sim	Sim	Sim	Sim
Lee DD *et al*.^*^	Sim	Sim	Sim	Sim	Sim	Não
Oliveira DSS *et al*.^**^	Sim	NI	Sim	Não	Sim	NI
Hamidi Y *et al*.^***^	Sim	Não	Não	Sim	Sim	Não
Zhang J *et al*.^*^	Sim	Sim	Sim	Sim	Sim	Sim
Harrison JD *et al*.^*^	Sim	Sim	Sim	Sim	Sim	Não
Ding Y *et al*.^**^	Sim	Sim	NI	Sim	Sim	NI
Scalvini S *et al*.^**^	Sim	Não	NI	Sim	Sim	NI

NI - não informado;

* - Baixo risco de viés;

** - Risco de viés incerto;

*** - Alto risco de viés.

**Quadro 3:** Avaliação do risco de viés de estudos de coorte com base na Escala de Newcastle-Ottawa Modificada, Botucatu, São Paulo, Brasil, 2024

Autor	Seleção				Comparabilidade	Desfechos			Total
	Representatividade da coorte exposta	Seleção da coorte não exposta	Determinação da exposição	Demonstração de que o resultado de interesse não estava presente no início do estudo	Comparabilidade das coortes com base no projeto ou análise	Avaliação do resultado	O acompanhamento foi longo o suficiente para que os resultados ocorressem	Adequação do acompanhamento das coortes	
Chen K *et al*.	C	a^*^	a^*^	a^*^	a^*^	a^*^	a^*^	a^*^	7^*^
Wu *et al*.	a^*^	a^*^	c	a^*^	a^*^	b^*^	a^*^	a^*^	7^*^

* *estrelas.*

Dos dez ensaios clínicos randomizados, cinco (50%) foram considerados com baixo risco de viés, quatro (40%), como risco de viés incerto, e um (10%), como estudo com alto risco de viés. Os estudos de coorte apresentaram sete estrelas, considerados de qualidade moderada.

## DISCUSSÃO

Como uma das ações da telenfermagem, o monitoramento é identificado como uma inovadora ferramenta no segmento do cuidado de saúde, que favorece a acessibilidade dos usuários aos serviços de saúde, e deve ser utilizado como um complemento da atenção à saúde, com o intuito de promover melhores condições de acesso, não de substituir ações já realizadas presencialmente nos serviços de saúde^(31)^.

A telenfermagem, no pós-operatório, mostra-se uma atividade fecunda, inovadora e desafiadora, que exige dos enfermeiros conhecimento científico, habilidade técnica e criatividade, e pode ser utilizada no processo de enfermagem, somada à oportunidade de promover o letramento em saúde. Nesse contexto, este estudo mostrou a predominância da aplicação do monitoramento como estratégia para a realização da assistência aos pacientes em pós-operatório no domicílio. Entre as principais intervenções, destacam-se a ligação telefônica^(1,21-27,29-30)^ e as mensagens de texto^(20,28)^. Os desfechos identificados nos estudos são diversos e divergem também entre os grupos de cirurgias e pacientes avaliados.

Como desfechos relacionados à experiência cirúrgica, a telenfermagem favoreceu o autocuidado nas dimensões habilidades gerais de autocuidado, habilidades de autocuidado desenvolvidas e habilidades de autocuidado desenvolvidas com estado de saúde ruim e pontuações totais na Escala de Agência de Autocuidado de acordo com a *Appraisal of Self-Care Agency Scale* ^(21)^. Em relação à retomada das atividades de vida diária, as intervenções telefônicas trouxeram resultados significativos na autonomia funcional dos pacientes idosos, medidos pelo *Functional Autonomy Measurement System* (SMAF)^(25)^. Ensaio clínico randomizado, realizado na China, incluindo 100 pacientes no pós-operatório de cirurgia de cabeça e pescoço, comparou o monitoramento telefônico *versus* o tradicional. Os resultados mostram que a telenfermagem aumentou os conhecimentos e comportamentos de saúde (12,52±9,27 vs. 5,94±8,12; P<0,001^(21)^. Estudo conduzido, no Brasil, considera que abordagem telefônica é uma estratégia para promoção da saúde^(1)^_._

A telenfermagem mostra-se benéfica para diferentes desfechos de pacientes no pós-operatório de cirurgia de hérnia de disco lombar. Ensaio clínico randomizado com 33 pacientes no grupo intervenção e 34 no grupo controle objetivou comparar o efeito da caminhada apoiada por pedômetro por meio do monitoramento com ligação telefônica três semanas e após a conclusão do primeiro, segundo e terceiro meses do pós-operatório. Os desfechos avaliados foram os níveis de dor, incapacidade e qualidade de vida. Os resultados mostram que caminhar após cirurgia diminuiu os níveis de dor e incapacidade e aumentou a qualidade de vida^(24)^. Considerando o desfecho dor, os pacientes do grupo intervenção apresentaram redução nos níveis de dor sensório-perceptual, dor momentânea e dor verbal mensurados pelo *The Short Form McGill Pain Questionnaire* (SF-MPQ) no segundo e terceiro meses pós-operatório^(24)^.

Outro estudo que avaliou qualidade de vida em 86 pacientes, submetidos à esofagectomia minimamente invasiva, mostrou que a telerreabilitação (reabilitação orientada por um aplicativo de mensagens denominado *WeChat* ^®^) contribui para a melhora na qualidade de vida global dos participantes segundo o *The European Organization for Research and Treatment of Cancer Quality of Life* (QoL) *Questionnaire Core* 30 (EORTC QLQ-C30)^(22)^.

Ensaio clínico randomizado multicêntrico, conduzido em oito hospitais canadenses, incluindo 905 pacientes no pós-operatório de diferentes cirurgias, comparou o cuidado virtual (ligação telefônica e monitoramento remoto automatizado) *versus* o cuidado presencial durante 31 dias. Verificou-se que os participantes do grupo de cuidados virtuais apresentaram menores níveis de dor moderada a intensa aos 7 (58,8%), 15 (48%) e 30 (35%) dias do pós-operatório. No entanto, não houve diferenças entre os grupos quando analisado o efeito das intervenções nas queixas dos pacientes e readmissões hospitalares^(24)^. Estudo piloto australiano, com seis meses de seguimento de 75 pacientes no pós-operatório de cirurgia colorretal, também sinaliza que a ligação telefônica é eficaz na melhora da qualidade de vida^(30)^.

A satisfação do usuário foi o desfecho avaliado em três estudos^(1,23,26)^. Pesquisa conduzida no Brasil, incluindo 81 pacientes submetidos à gastrocirurgia, mostrou que 87,5% dos pacientes avaliaram como excelente o atendimento pelo monitoramento via telefone *versus* 53,3% dos pacientes do grupo controle (atendimento presencial), com p=0,002^(1)^.

Estudo multicêntrico, com a aplicação do *Patient Satisfaction Questionnaire* - 18 (PSQ-18), objetivou determinar se a satisfação do paciente em encontros virtuais não é inferior à satisfação em encontros presenciais tradicionais para pacientes submetidos à cirurgia reconstrutiva de prolapso de órgãos pélvicos. Foram incluídas no estudo 52 participantes randomizadas aleatoriamente em dois grupos. A pontuação média de satisfação do paciente foi de 80,7 ± 2,6 no grupo virtual e 81,2 ± 2,8 no grupo de consultório, consistente com não inferioridade. As taxas de complicações pós-operatórias foram de 31% no grupo virtual e 46% no grupo de consultório. Não houve diferenças significativas entre visitas ao consultório, visitas ao pronto-socorro e readmissões hospitalares até 90 dias após a cirurgia^(26)^.

Ensaio clínico randomizado, desenvolvido na China, com 103 participantes submetidos à colostomia, evidenciou que não houve diferença significativa entre os grupos de controle e intervenção no nível de satisfação inicial. No entanto, um mês e três meses após a alta, o grupo intervenção apresentou satisfação significativamente maior^(23)^.

Outros estudos^(23,26)^ que avaliaram a satisfação do paciente em diferentes contextos cirúrgicos apresentaram resultados semelhantes em relação ao atendimento presencial e ao remoto, contudo o fator tempo foi melhor avaliado pelos participantes. O tempo despendido pelos usuários para se dirigirem ao consultório, o tempo de espera até o atendimento ser iniciado e o tempo de retorno para o seu domicílio foram as principais insatisfações relatadas. Como resultado, a duração do atendimento remoto, excluindo o tempo de viagem, foi quase um terço inferior comparado ao grupo com atendimento presencial^(23,26)^. Estudo com foco no paciente oncológico evidenciou que o monitoramento telefônico proporcionou maior satisfação dos pacientes no grupo intervenção, demonstrando o real impacto desse processo no cuidado de pacientes oncológicos^(1)^.

Corroborando o estudo, a literatura aponta outros benefícios da telenfermagem associados à satisfação, como redução de barreiras quanto ao acesso aos serviços, aconselhamento oportuno e cuidados de enfermagem prestados por enfermeiros na modalidade remota, favorecendo aos usuários a capacitação para exercer controle sobre seu processo de recuperação^(22-23,25,32-33)^.

Considera-se um desfecho ainda controverso, que necessita ser melhor estudado, a contribuição da telenfermagem para a utilização adequada das unidades de urgência pelos pacientes no pós-operatório. Estudo brasileiro, com monitoramento de pacientes com câncer de esôfago e estômago por nove meses no pós-operatório, verificou que, entre os 40 pacientes do grupo controle e 41 pacientes do grupo intervenção, não houve redução do número de admissões no serviço de emergência^(1)^. Diferentemente, estudo desenvolvido no pós-operatório de prostatectomia evidenciou que os pacientes do grupo intervenção apresentaram redução na busca por serviços de urgência, quando comparados com o grupo controle^(34)^. Estudo piloto australiano, com seis meses de seguimento de 75 pacientes no pós-operatório de cirurgia colorretal, também sinaliza que a ligação telefônica é eficaz na redução das taxas de reinternações hospitalares e de procura pelos serviços de emergência^(30)^.

No presente estudo, destaca-se a especialidade da gastrocirurgia, que foi objeto de estudo em seis pesquisas com foco na telenfermagem^(1,20,22-23,28,30)^, dos quais três^(20,23,28)^ abordaram as cirurgias para realização de colostomia/jejunostomia. Em relação aos cuidados com ostomias, ensaio clínico realizado no Irã, com 64 pacientes, constatou que, após um mês da intervenção, com ligação telefônica e envios de SMS, verificou-se diferença significativa entre os dois grupos na comparação do ajuste da estomia e suas dimensões (p<0,001)^(28)^. Consonante com esse resultado, ensaio clínico randomizado, com 103 participantes, realizado na China, avaliou os pacientes em relação à Escala de Ajuste de Ostomia (*Ostomy Adjustment Scale*) (OAS) e Escala de Autoeficácia para Estoma (*Stoma Self-Efficacy Scale*) (SSES). Os participantes do grupo intervenção, no terceiro mês, tiveram ajuste de ostomia significativamente melhor e maior autoeficácia para estoma, em comparação com o grupo controle (p=0,006)^(23)^.

Quanto aos aspectos relacionados à saúde mental, estudo avaliou a depressão e a ansiedade três meses após a alta do pós-operatório de enterostomia utilizando a estratégia *WeChat^®^* (mensagens de texto). Por meio dos instrumentos de autoavaliação de ansiedade (*Self-Rating Anxiety Scale* (SAS)) (p=0,017) e depressão (*Self-Rating Depression Scale* (SDS)) (p=0,012), os resultados mostram que o grupo intervenção apresentou diferença estatisticamente significativa na pontuação SAS e SDS, quando comparados ao grupo controle^(20)^. Investigação que analisou, durante seis meses, os efeitos de um monitoramento em 119 pacientes, submetidos à colostomia permanente, verificou que os dois grupos apresentaram melhores notas de SAS e SDS na pós-intervenção, quando comparados à pré-intervenção (o grupo controle apresentou SAS pré-intervenção de 61,02±7,48 e 53,38±6,12 pós-intervenção e SDS pré-intervenção 60,29±7,21 e 52,07±4,26 pós-intervenção; o grupo de observação apresentou SAS pré-intervenção de 61,82±6,21 e 49,83±5,44 pós-intervenção e SDS pré-intervenção 59,87±6,44 e 47,96±4,79 pós-intervenção)^(35)^.

Uma lacuna identificada é a realização de estudos incluindo a população pediátrica. Um único estudo realizado com neonatos, uma coorte retrospectiva, com 308 pacientes, avaliou a descoloração, a erosão/ulceração e o crescimento excessivo de tecido (DET), comprovando que o grupo intervenção apresentou pontuação DET mais baixa (p=0,003) e necessitou de menos substituições de bolsas de colostomia três meses após a alta hospitalar (p=0,002), quando comparado ao grupo controle^(20)^.

Embora a telenfermagem mostra-se promissora para o monitoramento dos pacientes cirúrgicos, considera-se a necessidade de maior investigação, considerando diferentes cenários e rigor metodológico na execução dos estudos. Tal modalidade favorece o acesso à informação e, consequentemente, proporciona melhores resultados em saúde. A discussão entre pares possibilita um apoio entre especialistas, o que promove uma atuação cada vez mais assertiva, reduzindo o risco de danos decorrentes da assistência à saúde e favorecendo a aprendizagem significativa dos enfermeiros envolvidos^(36)^.

Destaca-se ainda a escassez de estudos conduzidos no Brasil, lacuna que deverá ser preenchida frente à Resolução COFEN n^o^ 696/2022^(8-9)^.

Por fim, observa-se que nenhum estudo foi direcionado aos pacientes no pós-operatório de cirurgia craniana, sinalizando a importância do estudo em desenvolvimento pelos pesquisadores que estão implementando um protocolo para monitoramento via chamada de vídeo direcionado aos pacientes adultos e pediátricos no pós-operatório de neurocirurgia.

### Limitações do estudo

Como limitação do estudo, destacamos a exclusão de estudos de revisão, qualitativos, editoriais, teses e dissertações. Também não realizamos o registro no *Open Science Framework* (OSF). Ademais, não se avaliou a qualidade do protocolo de monitoramento utilizado nos estudos, aspecto extremamente relevante que pode interferir nos desfechos.

### Contribuições para a área de enfermagem

A telenfermagem constitui uma tecnologia em saúde que pode fortalecer ainda mais a autonomia do profissional enfermeiro, destacando sua competência e relevância na equipe multiprofissional para tomada de decisões assertivas e contribuição significativa para promoção da saúde, prevenção e controle de agravos, seja no campo individual ou coletivo.

## CONCLUSÕES

Dos estudos identificados nesta revisão, nenhum apresentou aspectos negativos relacionados à utilização da telenfermagem e 11 (91,6%) identificaram pelo menos um desfecho com significância estatística no uso da telenfermagem *versus* atendimentos presenciais em serviços de saúde aos pacientes no período pós-operatório. Os desfechos identificados foram melhora dos níveis de incapacidade, autonomia e atividades da vida diária, habilidades do autocuidado, qualidade de vida, aprimoramento do conhecimento em saúde, satisfação do usuário, manejo de estomas, menor necessidade de cuidados agudos, menor taxa de eventos adversos e complicações pós-operatórias, redução dos níveis de dor, melhora nos escores de avaliação de ansiedade e depressão, de níveis de saúde mental e redução de custos.

A telenfermagem foi mais utilizada em países desenvolvidos, e o monitoramento foi a modalidade mais utilizada para a avaliação dos pacientes no pós-operatório.

Dessa maneira, observa-se um cenário promissor e relevante para atuação do enfermeiro, com a necessidade do desenvolvimento de novos estudos que incluam o desenvolvimento de protocolos que atendam à necessidade de cada serviço/especialidade e os aspectos benéficos na qualidade da assistência no período pós-operatório.
